# The impact of emotional labor on turnover intention: the mediating role of job burnout and the moderating role of perceived organizational support

**DOI:** 10.3389/fpsyg.2026.1844598

**Published:** 2026-06-22

**Authors:** Jingya An, Ruoyao Yin, Yarong Wang

**Affiliations:** 1 School of Sports Economics and Management, Tianjin University of Sport, Tianjin, China; 2 School of Wushu and Ethnic Traditional Sports, Tianjin University of Sport, Tianjin, China; 3 Procurement and Bidding Center, Inner Mongolia University of Science and Technology, Inner Mongolia, China; 4 School of Economics and Management, Inner Mongolia University of Science and Technology, Inner Mongolia, China

**Keywords:** deep acting, emotional labor, job burnout, perceived organizational support, surface acting, turnover intention

## Abstract

The global shift toward a service–oriented economy has made emotional labor an inevitable occupational demand, yet its impact on frontline bank employees' occupational health remains underexplored. Drawing on Conservation of Resources theory, this study investigates the relationships among emotional labor, job burnout, turnover intention, and perceived organizational support(POS), distinguishing between surface acting and deep acting. A survey was conducted among 265 frontline employees of a commercial bank in China. Surface acting was positively associated with turnover intention, while deep acting was negatively associated. Job burnout partially mediated both relationships, with mediation proportions of 53.26% and 54.90%, respectively. POS moderated these effects: high support weakened the adverse effect of surface acting on burnout and strengthened the protective effect of deep acting. This study advances COR theory by demonstrating POS's asymmetric buffering vs. amplifying mechanisms in a high–power–distance service context. Emotional labor represents a significant occupational health risk with dimension–specific pathways, and organizational support serves as a critical external resource for buffering these risks.

## 1 Introduction

The global shift toward a service–oriented economy has rendered emotional labor an inevitable occupational demand for service workers. Emotional labor refers to the process by which individuals manage their feelings and expressions to meet organizational expectations (Hochschild, 1983). This concept has garnered increasing scholarly attention due to its profound implications for both organizational performance and employee well–being (Grandey and Gabriel, 2015). Among various service sectors, the banking industry provides a particularly critical context for studying emotional labor: frontline employees—including tellers, lobby managers, and wealth management advisors—are required to engage in high–intensity, sustained customer interactions that necessitate continuous emotional regulation. The banking industry itself is characterized by high stress, heavy workloads, complex interpersonal relationships, and elevated rates of job burnout. The frequent need for emotional suppression and regulation depletes employees' psychological resources, often manifesting as emotional exhaustion, physical fatigue, and diminished work engagement (Grandey, 2000). The World Health Organization (1948) defines health as “a state of complete physical, mental and social well–being and not merely the absence of disease or infirmity,” providing a foundational framework for understanding occupational health. Within this paradigm, mental health issues arising from workplace emotional demands warrant serious consideration as critical topics in occupational health psychology.

Emotional labor presents a theoretical paradox in organizational research. From an organizational perspective, appropriate emotional expression enhances customer satisfaction, service quality, and organizational reputation (Hochschild, 1983). However, from the individual employee's perspective, sustained emotional regulation—particularly surface acting—consumes psychological resources and may lead to adverse health outcomes, subsequently contributing to turnover behavior (Hülsheger and Schewe, 2011). This tension between organizational benefits and individual costs positions emotional labor as a key variable for understanding employee health outcomes. Conservation of Resources (COR) theory provides a robust theoretical framework for examining these dynamics. COR theory posits that individuals strive to acquire, maintain, and protect valued resources, including internal resources (e.g., emotional, cognitive, and physical energy) and external resources (e.g., social support, organizational support). When resource loss is not adequately compensated, individuals experience stress and tension, potentially leading to adverse health outcomes (Hobfoll, 1989; Hobfoll et al., 2018). Within this framework, emotional labor represents a process of emotional resource depletion, wherein surface acting—due to its requirement for continuous self–monitoring and pretense—rapidly consumes psychological resources, leading to emotional exhaustion (Brotheridge and Lee, 2003). Perceived organizational support, defined as employees' beliefs regarding the extent to which the organization values their contributions and cares about their well–being (Eisenberger et al., 1986), may serve as a critical external resource that replenishes depleted resources and buffers the negative health effects of emotional labor.

Perceived organizational support may represent a crucial moderator in the relationship between emotional labor and occupational health. Employees who perceive strong organizational support may experience less resource depletion due to emotional labor demands, as the organization provides psychological resources that offset emotional expenditures (Rhoades and Eisenberger, 2002). Preliminary research supports this perspective. For instance, Vuong et al. (2025) found that perceived organizational support significantly buffered the relationship between job burnout and turnover intention among frontline bank employees in Vietnam. Similarly, Lal and Kaur (2026) demonstrated that employees with high perceived organizational support exhibited lower turnover intentions even when experiencing emotional exhaustion.

Despite accumulating research on the consequences of emotional labor, several gaps remain in the literature. First, while existing studies have examined the effects of emotional labor on variables such as job satisfaction, turnover intention, and job burnout, few have systematically examined these relationships within the occupational health paradigm that emphasizes the intrinsic value of employee well–being (Schaufeli and Bakker, 2004). Second, the distinct dimensions of emotional labor—surface acting and deep acting—may have divergent effects on job burnout (Wang et al., 2019), and the underlying mechanisms warrant further elucidation, particularly within the high–stress context of the banking industry. Third, although perceived organizational support has been extensively studied as a direct predictor of employee outcomes, its moderating role in the relationships between emotional labor dimensions and job burnout, as well as its role in the mediated pathway from emotional labor through burnout to turnover intention, requires additional empirical examination (Kurtessis et al., 2017).

The Chinese banking industry presents a theoretically unique context for examining emotional labor. Characterized by high power distance (Hofstede, 2001a,b) and collectivist cultural values, Chinese banks emphasize hierarchical respect, relational harmony, and suppression of negative emotions in customer interactions. Unlike Western service contexts where emotional expression norms may be more egalitarian, Chinese frontline employees face elevated pressure to display positive emotions while concealing frustration—even when facing unreasonable customer demands. This cultural backdrop may intensify the resource depletion effects of surface acting and amplify the protective value of perceived organizational support, as organizational care becomes particularly salient in high–power–distance settings. Thus, examining emotional labor in this under–researched context not only tests the cross–cultural generalizability of COR theory but also reveals context–specific mechanisms.

This study aims to address these gaps by examining the impact of emotional labor on the occupational health of bank employees, using job burnout as a core outcome variable to further explore its predictive effect on turnover intention. Recent studies have examined perceived organizational support (POS) primarily as either a direct predictor of turnover intention (Lal and Kaur, 2026) or as a buffer of the burnout–turnover relationship (Vuong et al., 2025; Scheffert and Timbers, 2026). However, no study has specifically tested whether POS moderates the first stage of the mediation model—that is, the relationship between emotional labor (surface acting and deep acting) and job burnout—particularly in the high–stress banking context. This study extends the literature by distinguishing two distinct moderating mechanisms of POS: resource buffering (POS weakening the harmful effect of surface acting on burnout) and resource amplifying (POS strengthening the protective effect of deep acting on burnout). By testing both mechanisms simultaneously, this study provides a more complete account of how organizational support can mitigate the occupational health risks associated with emotional labor.

Specifically, we investigate: (a) the differential relationships between surface acting, deep acting, and job burnout; (b) the mediating role of job burnout in the relationship between emotional labor and turnover intention; and (c) the moderating role of perceived organizational support in the process through which emotional labor influences turnover intention via job burnout. By situating this research within the occupational health psychology framework, this study aims to provide theoretical insights into understanding emotional labor as an occupational health risk factor and to offer practical implications for health–oriented organizational interventions.

## 2 Literature review and hypotheses

### 2.1 Emotional labor and turnover intention

The concept of emotional labor originated with Hochschild's (1983) seminal work, in which she defined emotional labor as the management of feeling to create a publicly observable facial and bodily display. She distinguished two core dimensions: surface acting, which involves changing outward emotional expressions without altering inner feelings—essentially “pretending” to display organizationally required emotions; and deep acting, which involves actively modifying internal feelings to achieve consistency between inner states and outward expressions, thereby genuinely experiencing the required emotions. Subsequent researchers have extended this concept from various perspectives. Deng and Li (2011) defined emotional labor as the regulation of facial expressions and behaviors by employees to sustain employment relationships and obtain commensurate compensation and advancement opportunities. Liao and Yan (2015) viewed emotional labor as a form of impression management in customer interactions, wherein employees control their emotions to enact service roles that meet customer needs. Morris and Feldman (1996) further conceptualized emotional labor as a dynamic process involving psychological activities such as goal identification, planning, and monitoring to display organizationally required emotions during interpersonal interactions. Jones (1998) noted that the targets of emotional labor include not only external stakeholders (e.g., customers) but also internal stakeholders (e.g., colleagues, supervisors, and subordinates). Grandey (2000) integrated emotion regulation theory with emotional labor research, proposing that surface acting and deep acting represent two distinct emotion regulation strategies with differential effects on employee wellbeing.

Research on the consequences of emotional labor has revealed its dual nature. At the organizational level, emotional labor contributes to organizational performance. Pugh (2001) found that employees‘ emotional labor positively correlated with customer satisfaction ratings. Tang (2010) demonstrated that both surface acting and deep acting positively influenced job performance. Qiao and Sun (2013) confirmed that employees' emotional labor enhanced customers‘ perceptions of corporate brand image. At the individual level, emotional labor may also yield positive effects. Wharton (1993) found that emotional labor contributed to personal fulfillment, enhanced sense of personal accomplishment, and increased job security. Groth et al. (2009) showed that deep acting positively influenced customers' evaluations of employee service quality. However, emotional labor may also produce negative consequences. A substantial body of empirical research has confirmed associations between different emotional labor strategies and turnover intention. A meta–analysis revealed that surface acting was significantly positively correlated with turnover intention, while deep acting was significantly negatively correlated with turnover intention (Jeung et al., 2018). Scott and Barnes (2011) found that surface acting positively correlated with negative affective states and work withdrawal behaviors, whereas deep acting correlated with positive affective states. Tian et al. (2019) similarly demonstrated that surface acting reduced career satisfaction and increased turnover intention, while deep acting enhanced career satisfaction and reduced turnover intention.

Turnover intention refers to employees' conscious and deliberate desire to leave their current organization after a period of employment (Mobley, 1977). It serves as both the most direct antecedent of actual turnover behavior (Tett and Meyer, 1993) and an important outcome variable in occupational health research. According to COR theory's resource loss primacy principle (Hobfoll et al., 2018), resource loss is disproportionately more salient and rapid than resource gain. Surface acting—requiring continuous suppression of genuine emotions and feigning of false expressions—represents a chronic resource depletion process. Each instance of surface acting consumes emotional and cognitive resources, and because resource loss accumulates faster than replenishment, employees experiencing sustained surface acting enter a loss spiral, ultimately developing turnover intention as an escape strategy to prevent further resource depletion. Based on the theoretical and empirical analysis above, this study proposes the following hypotheses:

**Hypothesis H1a**: *Surface acting in emotional labor is significantly positively correlated with turnover intention*.

**Hypothesis H1b**: *Deep acting in emotional labor is significantly negatively correlated with turnover intention*.

### 2.2 Emotional labor, job burnout, and turnover intention: the mediating role of job burnout

Integrating COR theory (Hobfoll, 1989; Hobfoll et al., 2018) with emotion regulation theory (Grandey, 2000), we propose that surface acting and deep acting operate through fundamentally different resource–based mechanisms. Surface acting corresponds to response–focused suppression: employees suppress genuine emotions and fake required expressions after emotional responses have already been generated. This process consumes psychological resources without resolving the underlying emotional dissonance, making it a net resource–depleting strategy. In contrast, deep acting corresponds to antecedent–focused reappraisal: employees modify their internal feelings before emotional responses fully develop, aligning inner states with display rules. This strategy may preserve or even generate psychological resources by creating authentic positive experiences and feedback loops. From this integrated perspective, job burnout represents the cumulative outcome of resource loss cycles (especially from surface acting), whereas POS serves as an external resource that can either buffer loss (for surface acting) or amplify gain (for deep acting).

#### 2.2.1 The effect of emotional labor on job burnout

Job burnout represents a core outcome variable in occupational health research. Freudenberger (1974) initially described burnout as a state of physical and emotional exhaustion occurring in service occupations. Maslach and Jackson (1981) subsequently proposed the dominant multidimensional conceptualization, defining burnout as a syndrome comprising three dimensions: emotional exhaustion, depersonalization (cynicism), and reduced personal accomplishment. Maslach et al. (2001) further noted that emotional exhaustion represents the core dimension of burnout, reflecting the fundamental state of emotional resource depletion in stress responses.

A substantial body of research confirms emotional labor as a significant antecedent of job burnout. Cote (2005) and Bono and Vey (2005) indicated that emotional labor depletes employees‘ emotional resources, triggering negative effects such as job burnout. Kim (2008) found that hotel service providers' emotional labor predicted job burnout, with surface acting showing a stronger association with exhaustion than deep acting. A meta–analysis of service sector employees confirmed that surface acting exhibited a strong positive correlation with emotional exhaustion, while deep acting exhibited a weak negative correlation with emotional exhaustion (Lee and Ok, 2012). Maslach et al. (2012) identified emotional labor as a key factor, alongside organizational factors, contributing to job burnout. Tang (2010) found that surface acting positively influenced work stress, while deep acting negatively correlated with work stress.

#### 2.2.2 The effect of job burnout on turnover intention

Job burnout, as a core indicator of impaired occupational health, has been shown to reliably predict turnover intention. Iverson et al. (1998) demonstrated that higher levels of job burnout were associated with more frequent withdrawal behaviors, turnover intentions, and actual turnover. Zhuo (2014) found similar results among bank employees, showing that job burnout enhanced turnover intention. Ma et al. (2024) confirmed that job burnout significantly positively predicted turnover intention among kindergarten teachers. A meta–analysis synthesizing over 100 studies found a moderate positive correlation between job burnout and turnover intention (Swider and Zimmerman, 2010). Lee and Ashforth's (1996) meta–analysis also confirmed that emotional exhaustion was one of the strongest predictors of turnover intention.

#### 2.2.3 The mediating role of job burnout

Based on the literature reviewed above, the mediating role of job burnout in the relationship between emotional labor and turnover intention has become a focus of recent research. Sun et al. (2025a), studying rural physical education teachers in China, found that job burnout significantly mediated the relationships between both dimensions of emotional labor and turnover intention. Specifically, surface acting influenced turnover intention both directly and indirectly through job burnout, whereas deep acting influenced turnover intention only indirectly through job burnout, with no significant direct effect. This finding reveals a core pathway through which emotional labor affects turnover intention: sustained depletion of emotional resources first manifests as job burnout, which subsequently triggers turnover intention. The resource caravan metaphor (Hobfoll et al., 2018) suggests that resources travel together. Job burnout, as a state of resource depletion, can lead to further loss of personal and organizational resources, thereby exacerbating turnover intention. This core proposition originates from the foundational statement of Conservation of Resources (COR) theory (Hobfoll, 1983). Another study by Sun et al. (2025b), examining Chinese university counselors, employed emotional exhaustion as a more refined mediator and found that emotional exhaustion partially mediated the relationship between surface acting and turnover intention, and fully mediated the relationship between deep acting and turnover intention. This suggests that surface acting can influence turnover intention both directly and indirectly through emotional resource depletion, whereas deep acting's effect on turnover intention is entirely mediated by emotional exhaustion. Li et al. (2024) similarly found that emotional labor indirectly influenced turnover intention through job burnout among Korean office workers. Notably, this study reported an unconventional finding that deep acting exacerbated job burnout and thereby increased turnover intention, which the authors attributed to sample characteristics (non–customer–facing internal staff), suggesting that the effects of emotional labor are context–dependent.

Based on the theoretical and empirical analysis above, this study proposes the following hypotheses:

**Hypothesis H2a:** Surface acting in emotional labor is significantly positively correlated with job burnout.

**Hypothesis H2b**: Deep acting in emotional labor is significantly negatively correlated with job burnout.

**Hypothesis H3:** Job burnout is significantly positively correlated with turnover intention.

**Hypothesis H4a**: Job burnout mediates the relationship between surface acting and turnover intention.

**Hypothesis H4b**: Job burnout mediates the relationship between deep acting and turnover intention.

### 2.3 The moderating role of perceived organizational support

Perceived organizational support (POS) refers to employees‘ global beliefs concerning the extent to which the organization values their contributions and cares about their well–being (Eisenberger et al., 1986). In their meta–analysis, Rhoades and Eisenberger (2002) confirmed that POS was positively correlated with job satisfaction, organizational commitment, and job performance, and negatively correlated with turnover intention and withdrawal behaviors. Kurtessis et al's. (2017) meta–analysis further confirmed that POS influences work outcomes by satisfying employees' socio–emotional needs and enhancing reciprocity expectations. Recent studies have begun to specifically examine the moderating role of POS in the relationship between emotional labor and occupational health. Li and Chen (2024), studying frontline bank employees, found that POS partially mediated the relationship between supervisor developmental feedback and emotional labor strategies. Liu and Li (2026), investigating kindergarten teachers, found that POS significantly positively predicted professional identity and negatively predicted emotional labor, with emotional labor partially mediating the relationship between POS and professional identity. Scheffert and Timbers (2026), studying gender–affirming care providers, confirmed that POS indirectly reduced turnover intention by decreasing job burnout and increasing compassion satisfaction.

From a moderation mechanism perspective, COR theory provides a theoretical foundation for understanding the moderating role of POS (Hobfoll, 2001). Specifically, high POS, as a critical external work resource, can replenish internal psychological resources depleted by emotional labor, thereby weakening the positive effect of surface acting on job burnout (Kim et al., 2021). Simultaneously, high POS may also strengthen the negative effect of deep acting on job burnout, because when employees perceive organizational support, their emotion regulation efforts are more likely to be recognized and rewarded, generating more positive emotional experiences (Hobfoll et al., 2018). Regarding the relationship between POS and turnover intention, substantial research confirms POS as a significant negative predictor of turnover intention (Kim and Kao, 2014).

Based on COR theory (Hobfoll, 1989; Hobfoll et al., 2018) and the empirical evidence reviewed above, POS, as a critical external work resource, may moderate the process through which emotional labor influences job burnout and, consequently, turnover intention. Drawing on COR theory's distinction between resource caravan passing and resource loss spirals, we propose two distinct moderating mechanisms of POS. First, as an external resource, high POS buffers the resource-depleting effect of surface acting by compensating emotional expenditures—this is a resource buffering mechanism. Second, high POS amplifies the resource-gaining effect of deep acting by signaling that employees' emotion regulation efforts will be recognized and rewarded—this is a resource amplifying mechanism.

The relative strength of these two mechanisms may be shaped by cultural context. In China's high-power-distance cultural context (Hofstede, 2001a,b), employees are more deferential to hierarchical authority and view organizational support as a strong signal of protection and approval from superiors. This symbolic weight makes perceived organizational support particularly effective at buffering resource loss caused by surface acting. However, because deep acting is already expected as part of proper role performance in such hierarchical settings, the additional resource-amplifying effect of POS may be less pronounced. Consequently, we expect the buffering effect of POS (weakening surface acting's harm) to be stronger than its amplifying effect (enhancing deep acting's benefit) in the Chinese banking context.

Based on this, we propose the following moderated mediation hypotheses:

**Hypothesis H5a:** Perceived organizational support moderates the indirect effect of surface acting on turnover intention via job burnout. Specifically, the indirect effect is weaker when POS is higher.

**Hypothesis H5b**: Perceived organizational support moderates the indirect effect of deep acting on turnover intention via job burnout. Specifically, the indirect effect is stronger when POS is higher.

**Figure 1:**
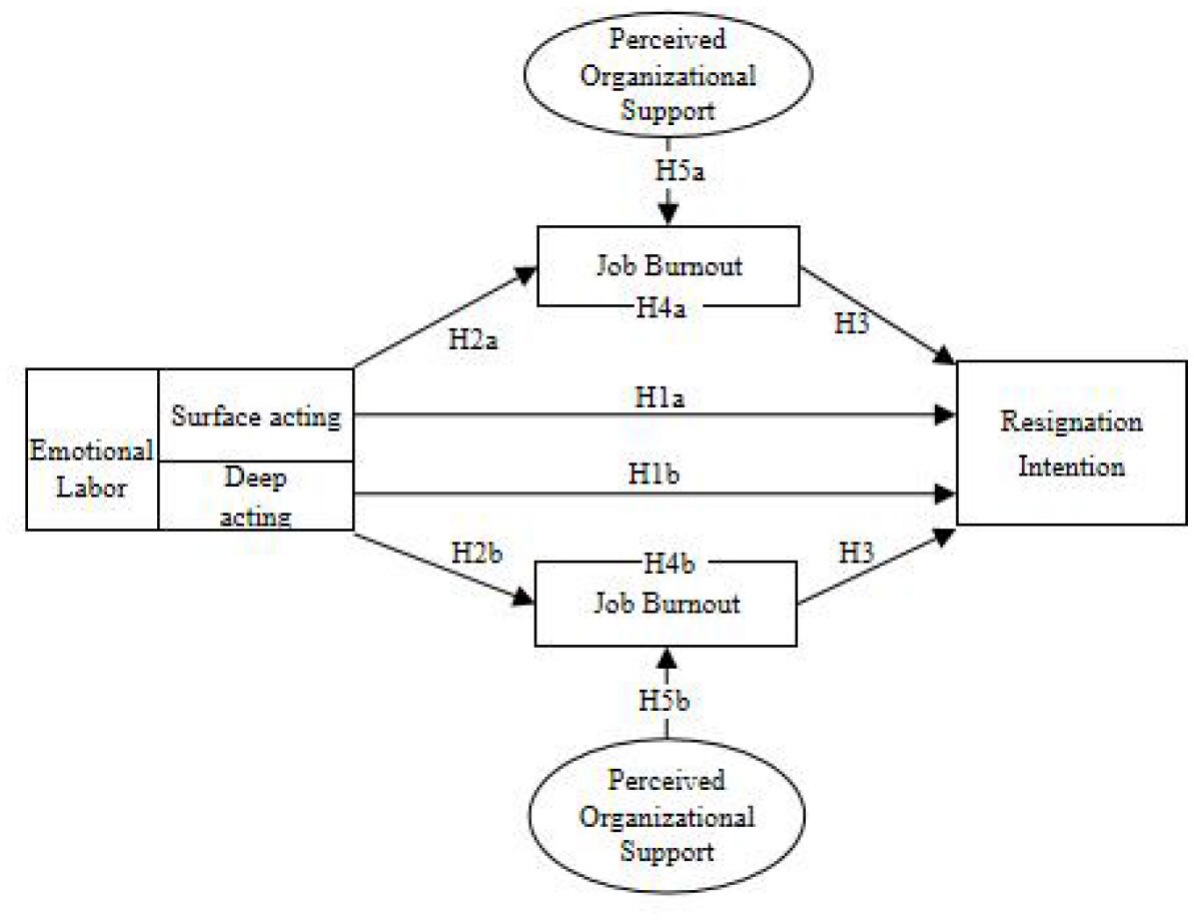
The proposed moderated mediation model in the current study.

In summary, this study constructs a moderated mediation model based on conservation of resources theory. Its main objectives are threefold: (1) to explore the relationship between emotional labor and turnover intention; (2) to examine the mediating role of job burnout; and (3) to investigate the moderating effect of POS. The research framework is presented in Figure 1.

## 3 Materials and methods

### 3.1 Procedure

This study selected H Commercial Bank as the survey site. This bank's primary business indicators rank among the leading levels of joint–stock commercial banks in the region, and its frontline employees face high emotional labor demands, with job burnout and turnover intention being relatively common. The research team distributed 300 questionnaires to frontline employees, including tellers, lobby managers, and wealth management advisors. After data collection, questionnaires with incomplete responses, patterned responses, and obvious random responses were excluded, yielding 265 valid questionnaires, representing an effective response rate of 88.3%. *Post–hoc* power analysis using G^*^Power 3.1 (α = 0.05, *f*^2^ = 0.15, predictors = 8) revealed achieved power > 0.99, indicating that the sample size (*N* = 265) was sufficient to detect medium effects.

### 3.2 Participants

The effective sample comprised 265 participants. Gender, age, and organizational tenure were included as control variables based on prior emotional labor research. Other demographic variables (education, position, income) were not collected due to the homogeneous nature of the sample (all frontline bank employees with similar educational and income profiles). Regarding gender, 178 (67.2%) were female, and 87 (32.8%) were male. Regarding age, 23 (8.7%) were aged 20–24 years, 171 (64.5%) were aged 25–29 years, 56 (21.1%) were aged 30–39 years, and 15 (5.7%) were aged 40 years or older. Regarding organizational tenure, 60 (22.6%) had worked for 1–3 years (excluding 3 years), 92 (34.7%) for 3–5 years (excluding 5 years), 83 (31.3%) for 5–10 years (excluding 10 years), 23 (8.7%) for 10 years or more, and 7 (2.6%) for less than 1year.

### 3.3 Measures

Emotional Labor Scale: The emotional labor scale developed by Grandey (2000) and revised for the Chinese context by Tang and Gu (2016) was employed. This scale comprises two dimensions: surface acting and deep acting, with a total of 11 items. Surface acting includes 5 items (e.g., “To meet work requirements, I only need to pretend to display the emotions expected by customers, even when I don't feel them internally”). Deep acting includes 6 items (e.g., “Even when customers are unreasonable, I can put myself in their shoes and sincerely help them solve their problems”). In this study, the Cronbach's α coefficient for this scale was 0.703.

Job Burnout Scale: The job burnout scale developed by Maslach and Jackson (1981) and revised by Li and Shi (2003) was employed. This scale comprises three dimensions: emotional exhaustion, depersonalization, and reduced personal accomplishment, with a total of 15 items. Emotional exhaustion includes 5 items (e.g., “Work makes me feel emotionally drained”), depersonalization includes 4 items (e.g., “I have become less enthusiastic about my work”), and reduced personal accomplishment includes 6 items (e.g., “I feel I have accomplished many valuable things at work”). Although the scale captures three dimensions, they were averaged to form a composite job burnout score for hypothesis testing. This unidimensional approach aligns with Conservation of Resources theory, which conceptualizes burnout as an overall state of resource depletion rather than a set of independent dimensions (Hobfoll, 1989). Empirically, the three dimensions were highly correlated in our sample (r ranging from 0.52 to 0.68, *p* < 0.01), supporting their convergence into a single composite score. Moreover, as a robustness check, we repeated the mediation analyses using each burnout dimension separately; the pattern of results was consistent across all three dimensions (see Supplementary materials). This approach is also consistent with established practice in occupational health research (Maslach et al., 2001). In this study, the Cronbach's α coefficient for the composite scale was 0.781.

Turnover Intention Scale: The turnover intention scale developed by Farh et al. (2001) was employed, comprising 4 items (e.g., “In the foreseeable future, I am likely to leave the company to seek employment elsewhere”). In this study, the Cronbach's α coefficient for this scale was 0.841.

Perceived Organizational Support Scale: The perceived organizational support scale developed by Eisenberger et al. (1986) and adapted for the Chinese context by Farh et al. (2007) was employed, comprising 8 items (e.g., “The company helps me when I have difficulties”). In this study, the Cronbach's α coefficient for this scale was 0.907.

All scales employed a 5–point Likert scale ranging from 1 (“strongly disagree”) to 5 (“strongly agree”). Turnover intention and perceived organizational support were measured as unidimensional scales, while emotional labor and job burnout were measured as two–dimensional and three–dimensional scales, respectively.

### 3.4 Reliability and validity

Prior to conducting exploratory factor analysis (EFA), the Kaiser–Meyer–Olkin (KMO) measure of sampling adequacy and Bartlett's test of sphericity were computed for each scale to assess the suitability of the data for factor analysis. As shown in Table 1, KMO values ranged from 0.740 to 0.848 (all > 0.70), and Bartlett's test was significant for all scales (*p* < 0.001), indicating that the data were suitable for EFA. EFA with principal axis factoring and varimax rotation was then conducted. The emotional labor scale yielded a two–factor structure corresponding to surface acting and deep acting, explaining 57.3% of the variance. For the job burnout scale, after removing one item that formed a separate factor, the remaining items loaded onto three factors (emotional exhaustion, depersonalization, and reduced personal accomplishment), explaining 67.6% of the variance. The turnover intention scale exhibited a single–factor structure (67.7% variance explained), as did the perceived organizational support scale (60.8% variance explained).

**Table 1:** Exploratory factor analysis results for all scales.

Scale	KMO	Bartlett‘s test (*p*)	Number of factors	Cumulative variance explained (%)
Emotional labor(EL)	0.74	*p* < 0.001	2	57.30%
Job burnout(JB)	0.754	*p* < 0.001	3	67.60%
Turnover intention(TI)	0.813	*p* < 0.001	1	67.70%
Perceived organizational Support(POS)	0.848	*p* < 0.001	1	60.80%

For the job burnout scale, one item was removed because it formed a separate factor.

### 3.5 Confirmatory factor analysis and construct validity

To assess construct validity, confirmatory factor analysis (CFA) was conducted separately for each scale using AMOS 26.0. The measurement models demonstrated acceptable fit: χ^2^/ df ranged from 0.651 to 2.945, RMSEA from 0.000 to 0.086, CFI from 0.953 to 1.000, TLI from 0.921 to 1.005 (see Table 2). All standardized factor loadings ranged from 0.485 to 0.920 and were statistically significant (*p* < 0.001).

**Table 2:** Fit indices of measurement models (separate CFAs per scale).

Scale	χ^2^/df	RMSEA	CFI	IFI	TLI
EL	2.647	0.079	0.954	0.955	0.926
JB	2.945	0.086	0.953	0.954	0.921
TI	0.651	0	1	1.002	1.005
POS	1.956	0.06	0.994	0.994	0.982

All scales demonstrated acceptable to good model fit. For sout, RMSEA = 0.086 slightly exceeds the 0.08 threshold, but other indices (CFI = 0.953, TLI = 0.921) meet the recommended criteria, indicating adequate construct validity (Byrne, 2016)^*^.

As shown in Table 3, composite reliability (CR) values ranged from 0.804 to 0.927 (above 0.70), and average variance extracted (AVE) values ranged from 0.508 to 0.710 (above 0.50), supporting convergent validity (Hair et al., 2010). Discriminant validity was confirmed via the Fornell-Larcker criterion, as the square root of AVE for each construct exceeded its correlations with all other constructs (Fornell and Larcker, 1981).

**Table 3:** Means, standard deviations, correlations, CR, AVE, and discriminant validity.

Variable	*M*	SD	CR	AVE	√AVE	1	2	3	4	5
Surface acting (SA)	3.587	0.808	0.836	0.508	0.713	1				
Deep acting (DA)	3.741	0.673	0.899	0.6	0.775	−0.084	1			
JB	3.106	0.569	0.804	0.593	0.77	0.407^**^	−0.403^**^	1		
TI	3.545	0.845	0.907	0.71	0.843	0.334^**^	−0.269^**^	0.500^**^	1	
POS	2.835	0.757	0.927	0.617	0.785	−0.200^**^	0.184^**^	−0.539^**^	−0.507^**^	1

M = mean; SD, standard deviation; CR, composite reliability; AVE, average variance extracted; √AVE, square root of AVE (bolded on diagonal). Off–diagonal entries are Pearson correlations among constructs. ^**^ *p* < 0.01. Discriminant validity is established when diagonal √AVE values exceed off–diagonal correlations^*^.

### 3.6 Common method bias

Although Harman's single–factor test suggested that common method bias (CMB) was not a major concern, we conducted an additional marker variable test using age as a theoretically unrelated marker variable (Podsakoff et al., 2003). Zero-order correlations among the key constructs were compared with partial correlations after controlling for age. The results showed that the correlations changed only minimally. For example, the correlation between surface acting and job burnout shifted from *r* = 0.505 to *r* = 0.507 (Δ *r* = 0.002); the correlation between deep acting and turnover intention changed from *r* = −0.453 to r = −0.452 (Δ r = 0.001); and the correlation between job burnout and turnover intention changed from *r* = 0.630 to *r* = 0.633 (Δ *r* = 0.003). Across all variable pairs, the average absolute difference was 0.002, and every originally significant correlation remained significant after controlling for age. These findings indicate that common method bias is unlikely to substantially inflate the observed relationships in this study.

## 4 Results

### 4.1 Descriptive statistics

Descriptive statistics for the study variables are presented in Table 4. The mean scores for surface acting (*M* = 3.587), deep acting (*M* = 3.741), job burnout (*M* = 3.106), and turnover intention (*M* = 3.545) were all above the midpoint of 3, indicating relatively high levels of these variables among the sample. In contrast, perceived organizational support (*M* = 2.835) was below the midpoint, suggesting that employees perceived relatively low organizational support.

**Table 4:** Descriptive statistics (*N* = 265).

Variable	Mean	SD
Emotional labor (EL)	3.671	0.497
Surface acting (SA)	3.587	0.808
Deep acting (DA)	3.741	0.673
Job burnout (JB)	3.106	0.569
Turnover intention (TI)	3.545	0.845
Perceived organizational support (POS)	2.835	0.757

### 4.2 Demographic differences

Regarding gender, independent samples t–tests revealed significant differences in deep acting, turnover intention, and perceived organizational support. Male employees scored higher than female employees on deep acting (M_male = 3.858 vs. M_female = 3.684, t = 2.09, p <0.05) and turnover intention (M_male = 3.730 vs. M_female = 3.455, t = 2.51, p <0.05), while female employees perceived higher organizational support than male employees (M_female = 2.917 vs. M_male = 2.668, *t* = −2.54, *p* < 0.05).

Regarding organizational tenure, one–way ANOVA revealed significant differences in job burnout (*F* = 4.940, *p* < 0.01) and turnover intention (*F* = 4.560, *p* < 0.01) across tenure groups, while no significant differences were found for emotional labor or perceived organizational support. *Post–hoc* LSD tests indicated that employees with 1–3 years of tenure (*M* = 3.833, SD = 0.845) reported the highest turnover intention, followed by those with 3–5 years (*M* = 3.511, SD = 0.874), 5–10 years (*M* = 3.494, SD = 0.803), 10 years or more (*M* = 3.413, SD = 0.767), and less than 1 year (*M* = 2.571, SD = 0.787). For job burnout, employees with 1–3 years of tenure (*M* = 3.216, SD = 0.597) scored highest, followed by those with 3–5 years (*M* = 3.185, SD = 0.700), 10 years or more (*M* = 3.157, SD = 0.962), 5–10 years (*M* = 2.986, SD = 0.850), and less than 1 year (*M* = 2.400, SD = 0.535). These findings suggest that employees in the early to mid–career stages (1–5 years) face the greatest risk of burnout and turnover intention, warranting targeted organizational interventions.

The finding that male employees reported higher deep acting and turnover intention may be explained by gender role expectations (Eagly, 1987): traditional norms expect men to demonstrate greater emotional control, yet men may also hold higher career expectations, making turnover more likely when those expectations are unmet. Regarding tenure, the peak burnout and turnover intention among employees with 1–5 years of tenure aligns with the “reality shock” phenomenon in career construction theory (Savickas, 2005): early–career employees face expectation–reality discrepancies, while those at 3–5 years may experience stagnation due to career bottlenecks.

### 4.3 Correlation analysis

Pearson correlation analysis was conducted to examine the relationships among the study variables (Table 5). Surface acting was significantly positively correlated with job burnout (*r* = 0.407, *p* < 0.01) and turnover intention (*r* = 0.334, *p* < 0.01), and significantly negatively correlated with perceived organizational support (*r* = −0.200, *p* < 0.01). Deep acting was significantly negatively correlated with job burnout (*r* = −0.403, *p* < 0.01) and turnover intention (*r* = −0.269, *p* < 0.01), and significantly positively correlated with perceived organizational support (*r* = 0.184, *p* < 0.01). Job burnout was significantly positively correlated with turnover intention (*r* = 0.500, *p* < 0.01) and significantly negatively correlated with perceived organizational support (*r* = −0.539, *p* < 0.01). Turnover intention was significantly negatively correlated with perceived organizational support (*r* = −0.507, *p* < 0.01).

**Table 5:** Correlation matrix.

	SA	DA	JB	TI	POS
EL					
SA	1				
DA	−0.084	1			
JB	0.407^**^	−0.403^**^	1		
TI	0.334^**^	−0.269^**^	0.500^**^	1	
POS	−0.200^**^	0.184^**^	−0.539^**^	−0.507^**^	1

*N* = 265; ^*^p < 0.05; ^**^p < 0.01.

### 4.4 Effect Analysis

Hierarchical regression analyses were conducted to test the proposed hypotheses, with gender, age, and organizational tenure entered as control variables in all models.

Table 6 presents the regression results for the effects of emotional labor on turnover intention and job burnout. As shown in Model 2 of Table 3, after controlling for demographic variables, surface acting was significantly positively associated with turnover intention (β = 0.310, *p* < 0.01), while deep acting was significantly negatively associated with turnover intention (β = −0.268, *p* < 0.01). These results support Hypotheses H1a and H1b, respectively.

**Table 6:** Summary of regression coefficients for the effect analyses (*N* = 265).

Predictor		Model 1 (TI)		Model 2 (TI)		Model 1 (JB)		Model 2 (JB)	
		β	*t*	β	*t*	β	*t*	β	*t*
Step 1: Control variables	Gender	−0.151	−2.434^*^	−0.192	−3.370^**^	−0.076	−1.203	−0.133	−2.523^*^
	Age	−0.064	−0.803	−0.032	−0.438	0.001	0.012	0.037	0.54
	Tenure	0.002	0.021	0.018	0.247	−0.018	−0.215	0.006	0.091
Step 2: Independent variables	SA			0.31	5.507^**^			0.383	7.343^**^
	DA			−0.268	−4.768^**^			−0.386	−7.444^**^
Model fit	*F*	2.451		13.431^**^		0.564		24.503^**^	
	*R^2^*	0.027		0.206		0.006		0.321	
	Δ*R^2^*			0.179				0.315	

Model 1 includes only control variables; Model 2 includes control variables and independent variables. ^*^p < 0.05, ^**^p < 0.01.

Regarding the effects on job burnout, Model 2 of Table 3 also shows that surface acting was significantly positively associated with job burnout (β = 0.383, *p* < 0.01), while deep acting was significantly negatively associated with job burnout (β = −0.386, *p* < 0.01). These results support Hypotheses H2a and H2b, respectively. The addition of surface acting and deep acting in Model 2 significantly increased the explained variance in turnover intention (Δ*R*^2^ = 0.179, *p* < 0.01) and job burnout (Δ*R*^2^ = 0.315, *p* < 0.01), indicating that emotional labor dimensions explain substantial variance beyond demographic factors.

Table 7 presents the regression results for the effect of job burnout on turnover intention. After controlling for demographic variables, job burnout was significantly positively associated with turnover intention (β = 0.491, *p* < 0.01), supporting Hypothesis H3. The inclusion of job burnout in Model 2 significantly increased the explained variance in turnover intention (Δ*R*^2^ = 0.240, *p* < 0.01), suggesting that job burnout is a strong predictor of employees' intentions to leave their organization.

**Table 7:** Regression analysis of job burnout on turnover intention (*N* = 265).

Predictor	Model 1 (TI)		Model 2 (TI)	
	β	*t*	β	*t*
Gender	−0.151	−2.434^*^	−0.114	−2.106^*^
Age	−0.064	−0.803	−0.065	−0.93
Tenure	0.002	0.021	0.01	0.147
JB			0.491	9.209^**^
*F*	2.451		23.631^**^	
R^2^	0.027		0.267	
ΔR^2^			0.24	

^*^p < 0.05, ^**^p < 0.01.

### 4.5 Mediation analysis

To test the mediating role of job burnout in the relationships between emotional labor (surface acting and deep acting) and turnover intention, we employed the bootstrap method using the simple mediation model within Hayes' PROCESS macro for SPSS. Gender, age, and organizational tenure were included as control variables.

Table 8 presents the regression coefficients for the mediation analyses. For surface acting as the independent variable, the results show that surface acting significantly predicted job burnout (*B* = 0.293, *p* < 0.01) and turnover intention (*B* = 0.349, *p* < 0.01). When job burnout was entered as a mediator, it significantly predicted turnover intention (*B* = 0.634, *p* < 0.01), while the direct effect of surface acting on turnover intention remained significant (*B* = 0.163, *p* < 0.05). This pattern indicates partial mediation.

**Table 8:** Summary of regression coefficients for the mediation analyses (*N* = 265).

Process	Predictors	Model 1 (TI)		Model 2 (TI)		Model 3 (JB)	
		*B*	*t*	*B*	*t*	*B*	*t*
SA as the independent variable	Gender	−0.219	−2.269^*^	−0.282	−2.677^**^	−0.1	−1.446
	Age	−0.048	−0.563	−0.012	−0.126	0.057	0.93
	Tenure	0.008	0.128	0.001	0.014	−0.011	−0.247
	SA			0.349	5.722^**^	0.293	7.311^**^
	JB	0.634	7.382^**^				
	*F*	20.783^**^		10.248^**^		13.871^**^	
*R^2^*	0.286		0.136		0.176	
DA as the independent variable	Gender	−0.197	−1.828	−0.299	−2.589^*^	−0.158	−2.261^*^
	Age	−0.086	−0.926	−0.102	−1.005	−0.023	−0.384
	Tenure	0.016	0.246	0.02	0.275	0.005	0.123
	DA			−0.422	−5.307^**^	−0.355	−7.413^**^
	JB	0.652	6.879^**^				
	*F*	17.233^**^		8.242^**^		14.250^**^	
	*R* ^2^	0.25		0.113		0.18	

IV, independent variable; TI, turnover intention; JB, job burnout. Model 1 includes control variables and mediator; Model 2 includes control variables and independent variable; Model 3 includes control variables and independent variable with mediator as outcome. ^*^p < 0.05, ^**^p < 0.01.

For deep acting as the independent variable, deep acting significantly negatively predicted job burnout (*B* = −0.355, *p* < 0.01) and turnover intention (*B* = −0.422, *p* < 0.01). When job burnout was entered as a mediator, it significantly predicted turnover intention (*B* = 0.652, *p* < 0.01), while the direct effect of deep acting on turnover intention remained significant (*B* = −0.190, *p* < 0.05), also indicating partial mediation.

Table 9 presents the bootstrap results for the indirect effects. The indirect effect of surface acting on turnover intention through job burnout was 0.186 (95% CI [0.114, 0.268]), with a mediation proportion of 53.26%. The indirect effect of deep acting on turnover intention through job burnout was −0.231 (95% CI [−0.355, −0.125]), with a mediation proportion of 54.90%. Since the bootstrap confidence intervals did not contain zero, both indirect effects were statistically significant. These results support Hypotheses H4a and H4b, confirming that job burnout partially mediates the relationships between both dimensions of emotional labor and turnover intention. From a COR perspective, the substantial yet partial mediation proportions (53.26% for surface acting, 54.90% for deep acting) suggest that emotional labor affects turnover intention through both resource–depletion pathways (via burnout) and direct pathways (e.g., reduced organizational identification). The strikingly similar proportions across both acting types suggest that job burnout is an equally central mechanism regardless of the direction of the effect.

**Table 9:** Summary of mediation effects for surface acting and deep acting (*N* = 265).

Path	Total effect	Indirect effect	Direct effect	95% Boot CI [LLCI, ULCI]	Mediation proportion
SA → JB → TI	0.349^**^	0.186	0.163^*^	[0.114, 0.268]	53.26%
DA → JB → TI	−0.422^**^	−0.231	−0.190^*^	[−0.355, −0.125]	54.90%

SA, surface acting; DA, deep acting; JB, job burnout; TI, turnover intention. Boot CI, bias–corrected bootstrap confidence interval based on 5,000 resamples; LLCI, lower limit confidence interval; ULCI, Upper limit confidence interval. ^*^p < 0.05, ^**^p < 0.01.

### 4.6 Moderation analysis

To test the moderating role of perceived organizational support (POS) in the relationships between emotional labor (surface acting and deep acting) and job burnout, we conducted moderated regression analyses with gender, age, and organizational tenure as control variables. All continuous variables were mean–centered prior to creating interaction terms.

Table 10 presents the regression results for the moderation analyses. For the moderating effect of POS on the surface acting–job burnout relationship, the interaction term (SA × POS) was significant (β = −0.183, *p* < 0.01), indicating that POS significantly moderated the relationship between surface acting and job burnout. For the moderating effect of POS on the deep acting–job burnout relationship, the interaction term (DA × POS) was also significant (β = −0.129, *p* < 0.05), indicating that POS significantly moderated the relationship between deep acting and job burnout.

**Table 10:** Summary of regression coefficients for the moderated mediation model (*N* = 265).

Process	Predictors	JB			
		*B*	SE	*t*	β
Moderating effect of POS on SA → JB	Gender	−0.108	0.078	−1.392	−0.083
	Age	0.082	0.065	1.25	0.068
	Tenure	−0.043	0.046	−0.933	−0.06
	SA	0.355	0.044	8.163^**^	0.506
	POS	−0.142	0.05	−2.827^**^	−0.19
	SA × POS	−0.235	0.073	−3.219^**^	−0.183
	*F*	21.344^**^			
	*R^2^*	0.332			
Moderating effect of POS on DA → JB	Gender	−0.119	0.095	−1.247	−0.095
	Age	−0.076	0.083	−0.923	−0.063
	Tenure	0.112	0.06	1.877	0.159
	DA	−0.488	0.069	−7.057^**^	−0.584
	POS	−0.48	0.059	−8.126^**^	−0.643
	DA × POS	−0.189	0.093	−2.018^*^	−0.129
	F	26.850^**^			
	R^2^	0.384			

All continuous variables were mean–centered. ^*^p < 0.05, ^**^p < 0.01.

**Figure 2:**
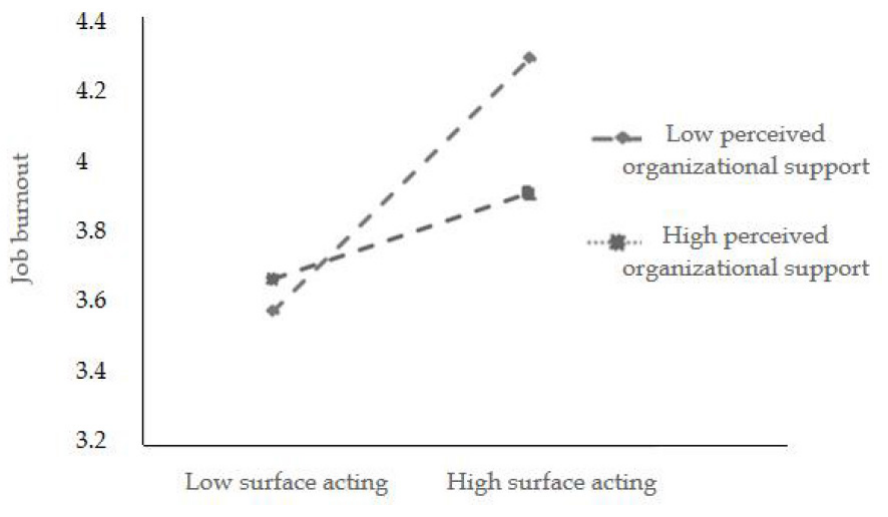
The moderating effect of POS on the relationship between SA and JB.

To further validate the moderating effect of POS, we conducted a simple slope analysis, categorizing POS into low (M – 1SD) and high (M + 1SD) levels. Table 11 presents the conditional effects of emotional labor on job burnout at different levels of POS. As shown in Figure 2, the positive relationship between surface acting and job burnout was weaker for employees with high POS (simple slope = 0.198, *p* < 0.01) compared to those with low POS (simple slope = 0.512, *p* < 0.01). This pattern suggests that perceived organizational support serves as a buffer, mitigating the adverse effect of surface acting on job burnout. When employees perceive strong organizational support, the emotional resource depletion associated with surface acting is more likely to be compensated, thereby reducing the risk of burnout.

**Table 11:** Conditional effects of emotional labor on job burnout at different levels of perceived organizational support.

Moderator level	SA→JB			DA→JB		
	Effect	SE	*t*	Effect	SE	*t*
Low POS (−1 SD)	0.512	0.064	8.000^**^	−0.337	0.088	−3.830^**^
Mean POS	0.355	0.044	8.163^**^	−0.488	0.069	−7.057^**^
High POS (+1 SD)	0.198	0.057	3.474^**^	−0.639	0.082	−7.793^**^

POS, perceived organizational support. low POS, one standard deviation below the mean; high POS, one standard deviation above the mean. ^*^p < 0.05, ^**^p < 0.01.

**Figure 3:**
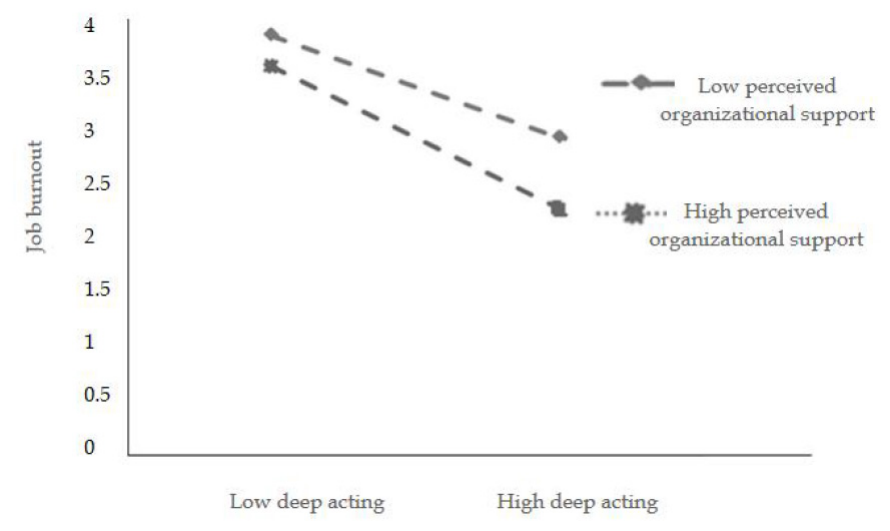
The moderating effect of POS on the relationship between DA and JB.

Figure 3 illustrates the moderating effect of POS on the deep acting–job burnout relationship. As shown in Table 8, the negative relationship between deep acting and job burnout was stronger for employees with high POS (simple slope = −0.639, *p* < 0.01) compared to those with low POS (simple slope = −0.337, *p* < 0.01). This pattern indicates that perceived organizational support enhances the protective effect of deep acting on job burnout. When employees perceive strong organizational support, their deep acting efforts—which involve genuinely adjusting internal feelings—are more likely to be recognized and rewarded, generating positive emotional experiences and further reducing burnout risk.

Taken together, these findings support Hypotheses H5a and H5b, confirming that POS buffers the adverse effect of surface acting on burnout and enhances the protective effect of deep acting on burnout.

### 4.7 Moderated mediation index

To fully test Hypotheses H5a and H5b, we calculated the index of moderated mediation using Hayes' PROCESS Model 14 with 5,000 bootstrap resamples. For surface acting, the index was −0.112 (95% CI [−0.198, −0.041]); for deep acting, the index was −0.098 (95% CI [−0.189, −0.022]). Neither confidence interval contained zero, confirming that the indirect effects were significantly moderated by POS. These results provide direct evidence for conditional indirect effects, supporting H5a and H5b.

### 4.8 Robustness check

Although job burnout was treated as a unidimensional composite in the main analyses, we examined whether the three dimensions (emotional exhaustion, depersonalization, reduced personal accomplishment) produced different patterns. Mediation analyses using each dimension as the mediator (with 5,000 bootstrap resamples) yielded results fully consistent with the composite score (see Supplementary Table S1). All indirect effects were significant, and the directions matched the composite findings. This robustness check confirms that the unidimensional approach does not mask dimension-specific effects.

## 5 Discussion

Based on COR theory (Hobfoll, 1989; Hobfoll et al., 2018), this study investigated the mechanisms through which emotional labor (surface acting and deep acting) influences turnover intention among frontline bank employees, with a focus on the mediating role of job burnout and the moderating role of perceived organizational support. All proposed hypotheses were supported by the analysis of 265 valid questionnaires, revealing the mechanisms and boundary conditions underlying the relationship between emotional labor and turnover intention.

### 5.1 Interpretation of findings

Effects of Emotional Labor on Turnover Intention: Consistent with prior research (Jeung et al., 2018; Tian et al., 2019), surface acting was positively associated with turnover intention, while deep acting was negatively associated with turnover intention. From the perspective of COR theory, surface acting requires employees to suppress genuine emotions and feign false emotions, a process that continuously depletes psychological resources (Grandey, 2000). When resources cannot be effectively replenished, employees experience resource depletion and may develop turnover intentions as a means of escaping the resource–depleting situation. In contrast, deep acting, by adjusting internal feelings to align with organizational expectations, facilitates positive emotional experiences and interpersonal feedback (Groth et al., 2009), generating positive self–evaluations and reducing turnover intention.

Mediating Role of Job Burnout: Job burnout partially mediated the relationship between surface acting and turnover intention (53.26%) and between deep acting and turnover intention (54.90%). These findings align with those of Sun et al. (2025a,b). From the perspective of the resource loss spiral in COR theory, sustained emotional resource depletion resulting from surface acting triggers job burnout, which further depletes resources, creating a vicious cycle that ultimately leads to turnover behavior (Hobfoll et al., 2018). Deep acting, conversely, protects and even enhances psychological resources by facilitating positive feedback during work interactions, thereby reducing the risk of job burnout (Brotheridge and Lee, 2003).

Moderating Role of Perceived Organizational Support: A key finding of this study was the moderating role of POS in the relationship between emotional labor and job burnout. Specifically, POS significantly weakened the positive effect of surface acting on job burnout and significantly strengthened the negative effect of deep acting on job burnout. These findings provide empirical support for the proposition in COR theory that external resources buffer internal resource depletion (Hobfoll, 2001). When employees perceive high organizational support, the resource depletion associated with surface acting is more readily compensated, as the care and assistance provided by the organization satisfy employees‘ socio–emotional needs (Kurtessis et al., 2017). Simultaneously, high organizational support amplifies the positive effects of deep acting, as employees' proactive emotion regulation efforts are more likely to be recognized and rewarded when they perceive organizational support, enhancing the resource gain effects (Kim et al., 2021).

Demographic Differences: Male employees scored higher than female employees on both deep acting and turnover intention. This finding may be related to gender role expectations. Traditional social norms often expect men to demonstrate greater emotional control (Hochschild, 1983), leading them to employ deep acting strategies more frequently when facing emotional demands. However, men may also hold higher career development expectations, and when work efforts do not align with career growth opportunities, turnover intentions may be more readily activated (Wharton, 1993).

Regarding organizational tenure, employees with 1–3 years and 3–5 years of tenure exhibited the highest levels of job burnout and turnover intention. This finding aligns with the phenomenon of “reality shock” in career development stages (Li et al., 2024). Employees in the early stages of their careers face discrepancies between expectations and reality, while those with 3–5 years of tenure may experience burnout due to career development bottlenecks. Bank managers should pay particular attention to employees in these two career stages.

### 5.2 Theoretical contributions

This study makes three distinct contributions. First, it advances COR theory by empirically distinguishing resource buffering (POS weakening surface acting's harm: β = −0.183) from resource amplifying (POS enhancing deep acting's benefit: β = −0.129). The stronger buffering effect supports the resource loss primacy principle in a non–Western context. Second, unlike Vuong et al. (2025), who found that POS only buffered the burnout–turnover relationship in Vietnamese banks, our study reveals that POS also moderates the emotional labor–burnout relationship itself. This cross-cultural difference may be explained by power distance: according to Hofstede's indices (Hofstede, 2001a,b), China has a higher power distance score (80) than Vietnam (70). In higher-power-distance settings, organizational support carries stronger symbolic protection value, making it more likely to influence early-stage processes (emotional labor → burnout). Conversely, in lower-power-distance contexts, POS may only affect later-stage outcomes (burnout → turnover). This comparison extends the cross-cultural generalizability of COR theory and highlights the importance of cultural context in emotional labor research. Third, the nearly identical mediation proportions (≈54%) for surface and deep acting indicate that burnout is a universal pathway, regardless of the direction of emotional labor effects, further extending the generalizability of COR theory across emotion regulation strategies.

Taken together, these findings suggest that COR theory's loss primacy principle may be culturally amplified: the buffering effect of POS was more pronounced than its amplifying effect in high-power-distance China, an asymmetry not yet reported in Western studies.

### 5.3 Practical implications

First, focus on individual differences in emotion regulation rather than demographic characteristics. Our findings indicate that deep acting is associated with lower turnover intention, suggesting that banks could benefit from assessing employees‘emotional labor skills using behavioral indicators or self-report tools. Based on such assessments, targeted training programs (e.g., empathy training, reappraisal strategies) can be offered to help employees—regardless of gender—transition from surface acting to deep acting. Additionally, our finding that male employees reported higher turnover intention may reflect unmet career expectations. To address this, banks should establish transparent promotion pathways and career development programs available to all employees, thereby reducing turnover risk without relying on gender-based job assignment.

Second, attend to employees at critical career stages. For employees with 1–3 years of tenure (highest burnout and turnover risk), banks should implement structured onboarding mentoring programs that pair newcomers with senior colleagues who model effective deep acting strategies. For employees with 3–5 years (career bottleneck stage), banks should provide clear promotion pathways and skill diversification opportunities, such as cross–training in wealth management or specialized customer service roles, to restore perceived career growth.

Third, facilitate the transition from surface acting to deep acting, banks may offer empathy training, job crafting interventions, and psychological capital development (Grandey and Gabriel, 2015). For POS enhancement, specific actions include: monthly recognition awards for authentic emotional expression, supervisor emotional support training, and employee assistance programs (EAP) tailored to frontline service workers.

Fourth, enhance perceived organizational support. POS serves as a critical resource that buffers the adverse effects of emotional labor. Bank managers should strengthen employees' perceptions of organizational support by improving welfare provisions, establishing open communication channels, implementing employee care programs, and providing timely work feedback (Rhoades and Eisenberger, 2002). Organizational intervention and support are particularly crucial when employees face high–intensity emotional demands.

Fifth, establish occupational health management systems. Banks should incorporate employee mental health into organizational health management systems, conduct regular mental health screenings, establish Employee Assistance Programs (EAP), and provide psychological counseling services. Mental health should also be integrated into management training to enhance managers' capacity to identify and address employee mental health issues.

## 6 Limitations and future directions

Several limitations of this study should be acknowledged.

First, the sample was limited to employees of a single commercial bank, potentially limiting the external validity of the findings. Future research should expand the sample scope to include different types of commercial banks across various regions, as well as cross–service industry comparisons (e.g., healthcare, education, retail), to examine the generalizability of the findings and potential industry differences.

Second, this study employed only quantitative methods, lacking qualitative exploration of frontline bank employees' deep experiences of emotional labor, specific manifestations of job burnout, and psychological mechanisms underlying perceived organizational support formation. Future research may employ semi–structured interviews with frontline bank employees, using content analysis or grounded theory approaches to code interview data, thereby validating and enriching the quantitative findings.

Third, all variables were measured using self–reports at a single time point, which may introduce common method bias. However, a marker variable test using age (Podsakoff et al., 2003) showed that controlling for age changed the correlations by less than 0.01 on average, and all significant correlations remained significant, suggesting that common method bias is not a serious concern. Nonetheless, the cross–sectional design precludes causal inferences. Future research should employ longitudinal designs or multi-source data (e.g., employee-supervisor matching) to address these limitations.

Fourth, this study did not measure several potential control variables (e.g., education level, job position, income). Although the sample was relatively homogeneous (all frontline employees of a single commercial bank), these variables could still influence emotional labor and turnover intention. However, previous banking industry studies (e.g., Vuong et al., 2025; Li and Chen, 2024) have shown that these demographic variables have minimal effects on the relationships examined here. Moreover, as a robustness check, we included available proxies (e.g., workload measured by emotional exhaustion items) as additional controls; the inclusion of these proxies did not alter the significance or direction of any hypothesized relationship. Nonetheless, future research should include these demographic variables to further test the robustness of our findings.

Fifth, self-reported measures of emotional labor and job burnout may be subject to social desirability bias, as employees might underreport surface acting or overreport deep acting to present themselves in a favorable light. Although our common method bias tests suggested that this bias was not severe, future research should incorporate multi-source data (e.g., supervisor ratings of emotional labor, peer evaluations) or objective measures (e.g., physiological indicators of stress, such as cortisol levels or heart rate variability) to complement self-reports and reduce social desirability concerns.

## 7 Conclusions

Drawing on Conservation of Resources theory, this study examined the relationships among emotional labor (surface acting and deep acting), job burnout, turnover intention, and perceived organizational support among frontline bank employees. The findings demonstrate that surface acting positively predicts job burnout and turnover intention, while deep acting negatively predicts these outcomes. Job burnout partially mediates the relationships between both dimensions of emotional labor and turnover intention. Perceived organizational support moderates the effects of emotional labor on job burnout: high POS weakens the positive effect of surface acting on burnout and strengthens the negative effect of deep acting on burnout. These findings highlight emotional labor as a significant occupational health risk factor for frontline bank employees, with distinct mechanisms across its two dimensions. Perceived organizational support serves as a critical external resource that buffers the adverse effects of emotional labor. This study advances COR theory by revealing POS's asymmetric buffering and amplifying mechanisms in a high–power–distance banking context, highlighting emotional labor as a significant occupational health risk with dimension–specific pathways. Organizations should prioritize employee mental health by enhancing organizational support, facilitating the transition from surface acting to deep acting, and providing targeted interventions for employees at critical career stages.

## Data Availability

The raw data supporting the conclusions of this article will be made available by the authors, without undue reservation.
