# Supplementary material for: The impact of emotional labor on turnover intention: the mediating role of job burnout and the moderating role of perceived organizational support
Source: Front Psychol. 2026 Jun 22;17:1844598. doi: 10.3389/fpsyg.2026.1844598 (PMC13333432; doi:10.3389/fpsyg.2026.1844598)
Supplement: Supplementary file 2 [file Table_1.docx]

### Table S1. Robustness Check: Mediation Effects of Each Burnout Dimension

| **Independent Variable** | **Mediator** | **Indirect Effect** | **95% Bootstrap CI** | **Conclusion** |
| --- | --- | --- | --- | --- |
| Surface Acting (SA) | Emotional Exhaustion (EE) | 0.363 | [0.230, 0.523] | Significant |
| Surface Acting (SA) | Depersonalization (DP) | 0.391 | [0.254, 0.551] | Significant |
| Surface Acting (SA) | Reduced Personal Accomplishment (RPA) | 0.071 | [0.016, 0.169]¹ | Significant |
| Deep Acting (DA) | Emotional Exhaustion (EE) | -0.368 | [-0.547, -0.211] | Significant |
| Deep Acting (DA) | Depersonalization (DP) | -0.541 | [-0.731, -0.364] | Significant |
| Deep Acting (DA) | Reduced Personal Accomplishment (RPA) | -0.127 | [-0.260, -0.024] | Significant |

Note: The CI for SA→RPA→TI was derived from the product of the a and b path CIs (both negative), yielding a positive interval. All bootstrap CIs are bias-corrected with 5,000 resamples.
